# Social determinants of health associated with newly documented opioid‐related postpartum readmissions

**DOI:** 10.1002/pmf2.70481

**Published:** 2026-10-13

**Authors:** India Perez‐Urbano, Timothy Wen, Yongmei Huang, Alexander Friedman

**Affiliations:** ^1^ Department of Obstetrics and Gynecology Columbia University Irving Medical Center New York New York USA; ^2^ Division of Biomedical Informatics Department of Medicine University of California, San Diego La Jolla California USA; ^3^ Division of Maternal‐Fetal Medicine Department of Obstetrics Gynecology, and Reproductive Sciences University of California, San Diego La Jolla California USA

**Keywords:** opioid use disorder, postpartum readmission risk, social determinants of health

## Abstract

**Objective:**

Peripartum opioid‐related mortality has increased in recent years, and social determinants of health (SDOH) are associated with adverse maternal outcomes. We sought to identify risk factors (including SDOH risk factors) for 180‐day postpartum readmissions related to opioid use among individuals without a documented diagnosis of opioid use at delivery.

**Methods:**

We performed a retrospective cohort study using 2016–2021 data from the Nationwide Readmissions Database. Delivery hospitalizations and subsequent 180‐day postpartum readmissions with a new opioid‐use diagnosis were identified. Newly documented opioid use was defined as opioid dependence, abuse, or overdose documented at readmission without evidence of opioid use disorder at or prior to delivery. SDOH exposures were categorized into domains reflecting education/employment, physical environment, and social factors using International Classification of Diseases (ICD)‐10 codes. Temporal trends in opioid use–related postpartum readmissions were evaluated using joinpoint regression to estimate average annual percent change (AAPC) with 95% confidence intervals (CIs). Multivariable logistic regression was used to estimate associations between SDOH and 180‐day newly documented opioid use readmission, adjusting for demographic and clinical factors.

**Results:**

Among 10,278,674 delivery hospitalizations, 3129 individuals (3.0 per 10,000) experienced a postpartum readmission with newly documented opioid use. The rate of 180‐day postpartum opioid‐related readmission declined from 3.7 to 2.2 per 10,000 delivery hospitalizations between 2016 and 2021 (AAPC, −10.4%; 95% CI, −13.5% to −7.8%). In unadjusted models, education‐ or employment‐related SDOH (odds ratio [OR], 6.26; 95% CI, 2.70–14.51), SDOH related to the physical environment, housing, or economic circumstances (OR, 20.93; 95% CI, 14.81–29.57), and social SDOH (OR, 4.09; 95% CI, 2.51–6.65) were associated with opioid‐related readmission. In adjusted models, only SDOH related to the physical environment, housing, or economic circumstances remained independently associated with opioid‐related readmission (adjusted odds ratio [aOR], 4.87; 95% CI, 3.35–7.09).

**Conclusion:**

Although postpartum readmissions for newly documented opioid use have declined nationally, individuals experiencing adverse physical environment–related SDOH remain at significantly elevated risk. Early identification of structural vulnerability and linkage to supportive services may help reduce opioid‐related postpartum morbidity.

## 1 INTRODUCTION

Opioid use disorder (OUD) among women of reproductive age is highly prevalent in the United States and is associated with peripartum mortality, severe maternal morbidity, adverse neonatal outcomes, legal consequences, and substantial healthcare utilization [1, 2]. Peripartum drug overdose has become a leading contributor to maternal mortality, with the greatest increase in overdose‐related deaths occurring in the late postpartum period (43 days to 1 year after delivery) [3]. Between 2018 and 2021, postpartum overdose mortality doubled, largely driven by synthetic opioids such as fentanyl [4]. OUD is associated with nearly a threefold increased risk of all‐cause postpartum hospital readmission compared with patients without OUD, and these readmissions are more frequently complicated by severe maternal morbidity [5].

Social determinants of health (SDOH) are strongly linked to maternal morbidity and mortality [6, 7]. While OUD affects all populations, prior studies demonstrate that postpartum readmission among patients with OUD is associated with structural and sociodemographic vulnerabilities, including income level, insurance status, and race/ethnicity [8]. Substance use disorders (SUDs) remain widely underdiagnosed due to structural barriers including stigma, punitive policy environments, and inequitable access to care [9, 10]. The peripartum period is a time of increased interaction with healthcare systems, creating a critical opportunity for identification and intervention in previously unrecognized SUDs. However, little is known about the sociodemographic and clinical factors associated with opioid‐related complications among birthing individuals without a documented OUD diagnosis during pregnancy. A better understanding of these risk factors may provide actionable insights to reduce postpartum opioid‐related morbidity and mortality even among individuals without a prior OUD diagnosis. Given this knowledge gap, the objective of this study was to characterize risk factors associated with opioid‐related postpartum readmissions among birthing individuals without a documented pre‐existing diagnosis of OUD, and to describe associated temporal trends.

## 2 METHODS

### 2.1 Data source

This retrospective cohort study used data from the 2016–2021 Nationwide Readmissions Database (NRD), a publicly available all‐payer database maintained by the Healthcare Cost and Utilization Project (HCUP) [11]. The NRD includes nationally representative discharge‐level information from community hospitals across participating US states and allows for tracking of patient readmissions within a given state within the same calendar year using encrypted patient linkage identifiers. National estimates are generated using discharge‐level weights and strata supplied by HCUP. These weights are constructed against a target universe of all inpatient discharges from US community hospitals with strata defined by census region, urban–rural location, teaching status, and hospital bed size. HCUP applies the American Hospital Association (AHA) definition of a community hospital: all nonfederal, short‐term, general, and other specialty hospitals. This definition includes academic medical centers, public hospitals, and specialty obstetric and gynecologic hospitals. The NRD contains patient demographic characteristics, expected primary payer, hospital characteristics, and diagnoses and procedures coded using International Classification of Diseases (ICD)‐10‐clinical modification (CM)/Procedure Coding System (PCS). Because this study used de‐identified data, an exemption was obtained from the institutional review board.

### 2.2 Study population

Delivery hospitalizations among individuals aged 15–54 years were identified using ICD‐10 diagnosis and procedure codes that have been validated to capture more than 95% of delivery admissions [12]. The delivery hospitalization was designated as the index hospitalization. Patients were excluded if they had a diagnosis of OUD, opioid dependence, opioid abuse, or opioid poisoning documented during the delivery hospitalization or during any inpatient admission prior to the delivery hospitalization (Table S1). These exclusions were intended to restrict the cohort to individuals at risk for newly documented postpartum opioid‐related readmissions. Because complete ascertainment of readmissions within 180 days of delivery discharge was required for the primary outcome, the analytic cohort was restricted to delivery discharges occurring during the first 6 months of each calendar year, for which a full 180 days of subsequent in‐year observation is available. Predelivery exclusions were applied to all inpatient admissions observable within the same calendar year prior to the delivery admission. The length of this predelivery observation window therefore varies across patients, from essentially none for deliveries occurring in January to approximately 150 days for deliveries occurring in June, and is not a uniform 180‐day lookback. Only the first qualifying opioid‐related postpartum readmission was included for each patient.

### 2.3 Exposure definition: Social determinants of health

SDOH were identified from ICD‐10‐CM Z codes documented during the delivery hospitalization. These codes are assigned from information documented by clinicians, social workers, or case managers and do not derive from a standardized patient‐reported screening instrument. The education and employment domain included problems related to education and literacy (Z55), employment or unemployment (Z56), and occupational exposures (Z57). The physical environment, housing, and economic circumstances domain included problems related to the physical environment (Z58) and housing or economic circumstances (Z59). The social and psychosocial domain included problems related to the social environment, upbringing, primary support group, and other psychosocial circumstances (Z60 and Z62–Z65). Patients could be assigned to more than one domain. The exposure therefore represents documentation of a social need rather than direct measurement of the underlying social circumstance [13].

### 2.4 Outcome

The primary outcome was a 180‐day postpartum readmission related to opioids with a new diagnosis of opioid dependence, opioid abuse, or opioid poisoning, identified by ICD‐10‐CM codes (Table S1) [14, 15]. Only the first postpartum readmission meeting these criteria was included for analysis.

### 2.5 Covariates

Patient‐level characteristics included maternal age at delivery, expected primary payer, median household income quartile by patient ZIP code, and clinical comorbidities documented during the delivery hospitalization. Clinical comorbidities included mental health disorders, chronic hypertension, pregestational diabetes, obesity, severe maternal morbidity excluding transfusion, and pain conditions [16, 17]. Obstetric characteristics included preterm birth, cesarean delivery, third‐ and fourth‐degree laceration, and stillbirth. Hospital characteristics included teaching status, location, and bed size. Model covariates were selected a priori based on clinical relevance and possible associations with opioid exposure or postpartum readmission.

### 2.6 Statistical analysis

Weighted descriptive statistics were used to characterize the study population and estimate nationally representative rates of postpartum opioid‐related readmissions. Categorical variables were compared using Rao–Scott χ^2^ tests. Temporal trends in 180‐day opioid‐related postpartum readmissions were assessed using joinpoint regression, which employs log‐linear segmented regression to calculate the average annual percent change (AAPC) with 95% confidence intervals (CIs) [18, 19]. Unadjusted logistic regression models were first used to estimate associations between SDOH domains and newly documented postpartum opioid‐related readmission. Multivariable logistic regression models were then constructed to estimate adjusted odds ratios (aORs) and 95% CIs, controlling for demographic characteristics, comorbidities, obstetric factors, and hospital characteristics. Survey weights supplied by HCUP were applied to generate national estimates and account for the complex survey design of the NRD. All analyses were conducted using SAS 9.4 and the National Cancer Institute's Joinpoint Regression Program. In accordance with HCUP reporting standards, cell sizes of 10 or fewer are not reported.

## 3 RESULTS

Among 10,278,674 delivery hospitalizations, there were 3129 postpartum opioid‐related readmissions, corresponding to a rate of 3.0 per 10,000 delivery hospitalizations. The rate of 180‐day opioid‐related postpartum readmission declined over the study period from 3.7 per 10,000 deliveries in 2016 to 2.2 per 10,000 in 2021. Joinpoint regression demonstrated a statistically significant downward trend over time, with an AAPC of −10.4% (95% CI, −13.5% to −7.8%).

Demographic and clinical characteristics differed substantially between individuals with and without a postpartum opioid‐related readmission. In unadjusted analyses, Medicaid insurance was strongly associated with readmission compared with private insurance (OR, 5.61; 95% CI, 4.85–6.50), as was Medicare coverage (OR, 25.63; 95% CI, 19.74–33.28). In adjusted analyses, Medicaid insurance remained significantly associated with increased odds of opioid‐related readmission (aOR, 5.44; 95% CI, 4.65–6.36), as did Medicare (aOR, 14.31; 95% CI, 10.95–18.72) Table 1.

**TABLE 1:** Demographics and outcome models for 6‐month newly documented opioid readmission.

	No opioid‐related readmission	Opioid readmission	Unadjusted model		Adjusted model^a^	
	*N* (%)	*N* (%)	OR	95% CI	aOR	95% CI
Age category						
15–19 years old	521,847 (5.08%)	81 (2.59%)	0.42	0.3, 0.58	0.29	0.21, 0.4
20–24 years old	1,957,960 (19.05%)	607 (19.39%)	0.84	0.72, 0.97	0.68	0.58, 0.79
25–29 years old	2,971,054 (28.91%)	1101 (35.17%)	Reference		Reference	
30–34 years old	2,977,208 (28.97%)	843 (26.93%)	0.76	0.67, 0.87	0.96	0.84, 1.11
35–39 years old	1,512,404 (14.72%)	427 (13.66%)	0.76	0.64, 0.91	0.94	0.79, 1.11
40–54 years old	335,072 (3.26%)	71 (2.26%)	0.57	0.4, 0.8	0.62	0.44, 0.87
Payer						
Medicare	76,800 (0.75%)	190 (6.06%)	25.63	19.74, 33.28	14.31	10.95, 18.72
Medicaid	4,215,665 (41.03%)	2279 (72.82%)	5.61	4.85, 6.5	5.44	4.65, 6.36
Private insurance	5,522,305 (53.74%)	532 (16.99%)	Reference		Reference	
Self‐pay	146,349 (1.42%)	65 (2.06%)	4.58	3.05, 6.89	4.79	3.19, 7.19
Other	298,260 (2.9%)	59 (1.9%)	2.07	1.39, 3.07	1.98	1.34, 2.94
Median income quartile by ZIP code						
Income Quartile 1 (lowest)	2,744,374 (26.71%)	1147 (36.65%)	2.42	2.02, 2.9	1.25	1.03, 1.51
Income Quartile 2	2,684,858 (26.13%)	911 (29.1%)	1.96	1.63, 2.37	1.22	1.01, 1.48
Income Quartile 3	2,589,641 (25.2%)	667 (21.31%)	1.49	1.23, 1.81	1.1	0.9, 1.33
Income Quartile 4 (highest)	2,190,445 (21.32%)	378 (12.09%)	Reference		Reference	
Clinical Factors						
Obesity	1,070,036 (10.41%)	289 (9.23%)	0.87	0.74, 1.04	0.56	0.47, 0.67
Mental health condition	849,461 (8.27%)	1029 (32.89%)	5.44	4.85, 6.11	4.81	4.25, 5.45
Pregestational diabetes	128,438 (1.25%)	83 (2.65%)	2.15	1.59, 2.92	1.02	0.75, 1.39
Chronic hypertension	244,100 (2.38%)	128 (4.08%)	1.75	1.34, 2.29	1.18	0.9, 1.55
Delivery complications						
Stillbirth	53,070 (0.52%)	40 (1.28%)	2.5	1.64, 3.81	0.88	0.57, 1.37
Non‐transfusion severe morbidity	70,821 (0.69%)	153 (4.88%)	7.41	5.78, 9.49	3.76	2.89, 4.9
Preterm birth < 37 weeks	1,017,998 (9.91%)	901 (28.79%)	3.68	3.27, 4.13	2.67	2.35, 3.03
Cesarean delivery	3,302,063 (32.14%)	1321 (42.2%)	1.54	1.39, 1.72	1.24	1.11, 1.39
Third‐ and fourth‐degree laceration	142,212 (1.38%)	DS	0.16	0.06, 0.42	0.31	0.11, 0.84
Chronic pain conditions	176,963 (1.72%)	81 (2.58%)	1.51	1.01, 2.26	0.9	0.6, 1.34
Hospital location						
Metropolitan teaching	2,002,250 (19.49%)	655 (20.93%)	0.92	0.8, 1.05	0.84	0.73, 0.96
Metropolitan non‐teaching	7,328,832 (71.32%)	2197 (70.21%)	Reference		Reference	
Non‐metropolitan	944,462 (9.19%)	277 (8.86%)	0.9	0.72, 1.11	0.8	0.65, 1
SDOH codes						
Z55–Z57 (Education/ employment)	5,475 (0.05%)	DS	6.26	2.7, 14.51	2.08	0.89, 4.86
Z58–Z59 (Physical environment/ housing/ economic circumstances)	11,319 (0.11%)	71 (2.26%)	20.93	14.81, 29.57	4.87	3.35, 7.09
Z60, Z62–Z65 (Social factors)	23,463 (0.23%)	29 (0.93%)	4.09	2.51, 6.65	1.37	0.82, 2.26
Hospital Bed Size						
Small	1,788,183 (17.4%)	514 (16.43%)	0.94	0.81, 1.1	1.07	0.92, 1.25
Medium	2,856,124 (27.8%)	895 (28.6%)	1.03	0.9, 1.17	1.09	0.96, 1.24
Large	5,631,237 (54.8%)	1720 (54.97%)	Reference		Reference	
Year of delivery						
2016	1,787,548 (17.4%)	659 (21.07%)	Reference		Reference	
2017	1,741,778 (16.95%)	605 (19.33%)	0.94	0.79, 1.13	0.91	0.76, 1.09
2018	1,728,127 (16.82%)	564 (18.01%)	0.88	0.74, 1.06	0.84	0.71, 1
2019	1,702,421 (16.57%)	475 (15.18%)	0.76	0.63, 0.91	0.69	0.58, 0.83
2020	1,673,604 (16.29%)	464 (14.84%)	0.75	0.62, 0.92	0.67	0.55, 0.81
2021	1,642,067 (15.98%)	362 (11.58%)	0.6	0.49, 0.74	0.5	0.41, 0.62

Abbreviations: aOR, adjusted odds ratio; CI, confidence interval; HCUP, Healthcare Cost and Utilization Project; OR, odds ratio; SDOH, social determinants of health.

^a^ Adjusted for demographic, clinical, obstetric, and hospital characteristics. DS indicates data suppressed in accordance with HCUP requirements because the cell size was 10 or fewer.

Younger and older maternal ages were associated with lower odds of postpartum opioid‐related readmission relative to individuals aged 25–29 years. In adjusted models, individuals aged 15–19 years (aOR, 0.29; 95% CI, 0.21–0.40), 20–24 years (aOR, 0.68; 95% CI, 0.58–0.79), and 40–54 years (aOR, 0.62; 95% CI, 0.44–0.87) had lower odds of readmission compared with those aged 25–29 years. Compared with the highest ZIP‐code income quartile, the lowest (aOR, 1.25; 95% CI, 1.03–1.51) and second‐lowest (aOR, 1.22; 95% CI, 1.01–1.48) quartiles were associated with increased odds of readmission.

Clinical comorbidities were strongly associated with postpartum opioid‐related readmission. Individuals with a documented mental health condition during the delivery hospitalization had higher odds of opioid‐related readmission in adjusted analyses (aOR, 4.81; 95% CI, 4.25–5.45). Non‐transfusion severe maternal morbidity was also strongly associated with readmission (aOR, 3.76; 95% CI, 2.89–4.90), as was preterm birth (aOR, 2.67; 95% CI, 2.35–3.03). Obesity was associated with reduced odds of readmission (aOR, 0.56; 95% CI, 0.47–0.67). Among hospital characteristics, delivery at a metropolitan teaching hospital was associated with lower odds of readmission compared with delivery at a metropolitan non‐teaching hospital (aOR, 0.84; 95% CI, 0.73–0.96); the estimate for non‐metropolitan hospitals was aOR, 0.80 (95% CI, 0.65–1.00). Adjusted odds of postpartum opioid‐related readmission declined progressively over the study period, reaching approximately half the 2016 level by 2021 (aOR, 0.50; 95% CI, 0.41–0.62) (Figure 1).

**FIGURE 1:**
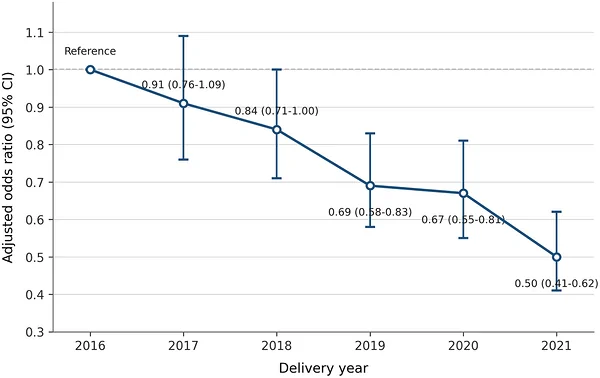
Adjusted odds of 180‐day postpartum readmission with a newly documented opioid‐related diagnosis by delivery year, 2016–2021. Points represent adjusted odds ratios and error bars represent 95% confidence intervals (CIs); 2016 was the reference year. Models were adjusted for demographic, clinical, and hospital characteristics.

SDOH were significantly associated with opioid‐related postpartum readmission in unadjusted analyses. Education/employment‐related SDOH codes were associated with increased odds of readmission (OR, 6.26; 95% CI, 2.70–14.51), as were physical environment/housing/economic circumstances‐related codes (OR, 20.93; 95% CI, 14.81–29.57) and social or psychosocial codes (OR, 4.09; 95% CI, 2.51–6.65). After adjustment for demographic, clinical, and hospital factors, only physical environment/housing/economic circumstances‐related SDOH remained independently associated with postpartum opioid‐related readmission (aOR, 4.87; 95% CI, 3.35–7.09). Education/employment‐related (aOR, 2.08; 95% CI, 0.89–4.86) and social or psychosocial SDOH codes (aOR, 1.37; 95% CI, 0.82–2.26) were not independently associated with readmission.

## 4 DISCUSSION

This study provides new insight into opioid‐related postpartum readmissions among individuals without a previously documented diagnosis of OUD. Our findings demonstrate that although postpartum readmissions with newly documented opioid diagnoses have declined over the study period, substantial risk remains among vulnerable populations. This decline may reflect expanded awareness of substance use disorders, increased perinatal screening efforts, and broader uptake of screening and treatment initiatives for substance use during pregnancy [20, 21, 22]. However, it may also reflect shifts in coding practices or increased diagnosis of opioid use during pregnancy, thereby reducing the number of cases identified for the first time postpartum.

Our analysis highlights that physical and environmental SDOH, as well as public insurance status, are independently associated with an increased likelihood of postpartum readmission with a newly documented opioid diagnosis. These findings are consistent with a growing body of evidence demonstrating that structural and material conditions such as housing instability, food insecurity, transportation barriers, and economic hardship are strongly associated with adverse maternal and perinatal outcomes [8, 23]. Physical environment–related SDOH may influence substance use risk through chronic stress, reduced access to care, limited treatment continuity, and increased exposure to environmental stressors. Addressing these structural factors requires policy‐level interventions and coordinated healthcare and community‐based strategies to reduce disparities.

Patients with substance use disorders are known to experience increased postpartum morbidity and readmission risk [5]. This elevated risk is compounded by higher rates of medical and psychiatric comorbidity. Prior work has demonstrated that, even after adjustment for comorbid conditions, opioid use remains more strongly associated with postpartum readmission compared with other substance exposures such as cocaine or amphetamines [24]. In our analysis, mental health disorders, preterm birth, and severe maternal morbidity were strongly associated with postpartum opioid‐related readmission. The relationship between mental health conditions and substance use is well documented, and integrated care models addressing both mental health and substance use have been associated with improved outcomes [25, 26].

Notably, we observed that Medicaid insurance was strongly associated with increased odds of postpartum opioid‐related readmission. This association likely reflects the intersection of socioeconomic disadvantage, barriers to consistent care access, and higher prevalence of untreated or undertreated substance use disorders in populations covered by public insurance [8]. These findings reinforce the importance of equitable access to comprehensive perinatal and postpartum care, particularly for individuals facing structural and socioeconomic vulnerabilities [23, 27].

This analysis should be interpreted in the context of several limitations. First, administrative data are subject to misclassification, under‐ascertainment, and limitations inherent to diagnostic coding. As such, some individuals categorized as having newly diagnosed postpartum opioid diagnoses may have had undiagnosed or undocumented OUD during pregnancy. Second, the NRD lacks clinical granularity regarding severity of substance use, treatment engagement, and social context beyond diagnostic codes. Third, linkage across calendar years is not possible, limiting follow‐up beyond the same calendar year. Fourth, the predelivery observation window in this analysis varies in length because the NRD does not permit patient linkage across calendar years. Individuals with an opioid‐related inpatient admission occurring earlier in pregnancy but before the start of the calendar year could not be identified and excluded. Some individuals classified here as having a newly diagnosed opioid‐related readmission may therefore have had a previously documented opioid‐related diagnosis that was not observable, which would inflate the estimated rate of the outcome. Such misclassification would bias the association with documented social risk only if it occurred differentially by exposure status, for which we have no direct evidence in either direction. Fifth, diagnostic codes for OUD in administrative data have been shown to have acceptable positive predictive value but only moderate sensitivity [14, 27, 28], and the proportion of preexisting OUD that went unidentified in this cohort cannot be quantified from these data. OUD managed exclusively in outpatient settings, including patients receiving office‐based medication for OUD, generates no inpatient record and is not observable in an inpatient database. Documentation may also be suppressed by clinician and patient concern regarding punitive consequences of recorded substance use in pregnancy. For all of these reasons, the outcome examined here is the first documentation of an opioid‐related diagnosis in the administrative data record and is not equivalent to incident OUD. Sixth, although the NRD is nationally representative, it is possible that the 6‐month subset of deliveries we analyzed is not. Seventh, ICD‐10 codes for SDOH are underutilized in clinical practice; therefore, adverse social conditions were likely under‐ascertained in this analysis.

Despite these limitations, the use of a large, nationally representative dataset provides substantial power and allows for robust population‐level estimates. A key strength of this study is its focus on postpartum individuals without a previously documented OUD diagnosis, a population for whom relatively little is known regarding risk factors and outcomes.

In conclusion, our findings suggest that individuals with concurrent mental health conditions and adverse SDOH, particularly those related to physical environment and housing, remain at disproportionately high risk for newly documented postpartum opioid‐related readmission. These findings underscore the importance of early identification of socioeconomic vulnerability and linkage to supportive services during pregnancy and the postpartum period. Addressing structural barriers and integrating behavioral health, social services, and obstetric care may help mitigate opioid‐related morbidity and improve postpartum outcomes.

## CONFLICT OF INTEREST STATEMENT

Alexander Friedman is supported by funding from the *Eunice Kennedy Shriver* National Institute of Child Health and Human Development (1R01HD104943); however, this funding was not used to support this research. Alexander Friedman has also served on an advisory board for Biogen and Sage. Timothy Wen serves as a consultant on the medical advisory board for Delfina, Inc. The other authors declare no conflicts of interest.

## FUNDING INFORMATION

The authors received no specific funding for this work.

## Supporting information



Supporting information
