# Supplementary material for: Social determinants of health associated with newly documented opioid‐related postpartum readmissions
Source: Pregnancy (Hoboken). 2026 Oct 13;2(6):e70481. doi: 10.1002/pmf2.70481 (PMC13659799; doi:10.1002/pmf2.70481)
Supplement: Supplementary file 1 [file PMF2-2-e70481-s001.docx]

| **Table 1. Demographics and outcome models for 6 month newly documented opioid readmission** | | | | | | |  |
| --- | --- | --- | --- | --- | --- | --- | --- |
|  | **No opioid-related readmission** | **Opioid readmission** | **Unadjusted model** | | **Adjusted model^1^** | |  |
|  | **N (%)** | **N (%)** | **OR** | **95%CI** | **aOR** | **95%CI** |  |
| **Age Category** |  |  |  |  |  |  |  |
| 15-19 years old | 521847(5.08%) | 81(2.59%) | 0.42 | 0.3, 0.58 | 0.29 | 0.21, 0.4 |  |
| 20-24 years old | 1957960(19.05%) | 607(19.39%) | 0.84 | 0.72, 0.97 | 0.68 | 0.58, 0.79 |  |
| 25-29 years old | 2971054(28.91%) | 1101(35.17%) | Reference | | Reference | |  |
| 30-34 years old | 2977208(28.97%) | 843(26.93%) | 0.76 | 0.67, 0.87 | 0.96 | 0.84, 1.11 |  |
| 35-39 years old | 1512404(14.72%) | 427(13.66%) | 0.76 | 0.64, 0.91 | 0.94 | 0.79, 1.11 |  |
| 40-54 years old | 335072(3.26%) | 71(2.26%) | 0.57 | 0.4, 0.8 | 0.62 | 0.44, 0.87 |  |
| **Payer** |  |  |  |  |  |  |  |
| Medicare | 76800(0.75%) | 190(6.06%) | 25.63 | 19.74, 33.28 | 14.31 | 10.95, 18.72 |  |
| Medicaid | 4215665(41.03%) | 2279(72.82%) | 5.61 | 4.85, 6.5 | 5.44 | 4.65, 6.36 |  |
| Private insurance | 5522305(53.74%) | 532(16.99%) | Reference | | Reference | |  |
| Self pay | 146349(1.42%) | 65(2.06%) | 4.58 | 3.05, 6.89 | 4.79 | 3.19, 7.19 |  |
| Other | 298260(2.9%) | 59(1.9%) | 2.07 | 1.39, 3.07 | 1.98 | 1.34, 2.94 |  |
| **Median Income Quartile by ZIP Code** |  |  |  |  |  |  |  |
| Income Quartile 1 (lowest) | 2744374(26.71%) | 1147(36.65%) | 2.42 | 2.02, 2.9 | 1.25 | 1.03, 1.51 |  |
| Income Quartile 2 | 2684858(26.13%) | 911(29.1%) | 1.96 | 1.63, 2.37 | 1.22 | 1.01, 1.48 |  |
| Income Quartile 3 | 2589641(25.2%) | 667(21.31%) | 1.49 | 1.23, 1.81 | 1.1 | 0.9, 1.33 |  |
| Income Quartile 4 (highest) | 2190445(21.32%) | 378(12.09%) | Reference | | Reference | |  |
| **Clinical Factors** |  |  |  |  |  |  |  |
| Obesity | 1070036(10.41%) | 289(9.23%) | 0.87 | 0.74, 1.04 | 0.56 | 0.47, 0.67 |  |
| Mental health condition | 849461(8.27%) | 1029(32.89%) | 5.44 | 4.85, 6.11 | 4.81 | 4.25, 5.45 |  |
| Pregestational diabetes | 128438(1.25%) | 83(2.65%) | 2.15 | 1.59, 2.92 | 1.02 | 0.75, 1.39 |  |
| Chronic hypertension | 244100(2.38%) | 128(4.08%) | 1.75 | 1.34, 2.29 | 1.18 | 0.9, 1.55 |  |
| **Delivery complications** |  |  |  |  |  |  |  |
| Stillbirth | 53070(0.52%) | 40(1.28%) | 2.5 | 1.64, 3.81 | 0.88 | 0.57, 1.37 |  |
| Non-transfusion severe morbidity | 70821(0.69%) | 153(4.88%) | 7.41 | 5.78, 9.49 | 3.76 | 2.89, 4.9 |  |
| Preterm birth <37 weeks | 1017998(9.91%) | 901(28.79%) | 3.68 | 3.27, 4.13 | 2.67 | 2.35, 3.03 |  |
| Cesarean delivery | 3302063(32.14%) | 1321(42.2%) | 1.54 | 1.39, 1.72 | 1.24 | 1.11, 1.39 |  |
| 3^rd^ or 4^th^ degree laceration | 142212(1.38%) | DS | 0.16 | 0.06, 0.42 | 0.31 | 0.11, 0.84 |  |
| Chronic pain conditions | 176963(1.72%) | 81(2.58%) | 1.51 | 1.01, 2.26 | 0.9 | 0.6, 1.34 |  |
| **Hospital location** |  |  |  |  |  |  |  |
| Metropolitan teaching | 2002250(19.49%) | 655(20.93%) | 0.92 | 0.8, 1.05 | 0.84 | 0.73, 0.96 |  |
| Metropolitan non-teaching | 7328832(71.32%) | 2197(70.21%) | Reference | | Reference | |  |
| Non-metropolitan | 944462(9.19%) | 277(8.86%) | 0.9 | 0.72, 1.11 | 0.8 | 0.65, 1 |  |
| **SDOH codes** |  |  |  |  |  |  |  |
| Z55-Z57 (Education/ employment) | 5475(0.05%) | DS | 6.26 | 2.7, 14.51 | 2.08 | 0.89, 4.86 |  |
| Z58-Z59 (Physical environment/ housing/ economic circumstances) | 11319(0.11%) | 71(2.26%) | 20.93 | 14.81, 29.57 | 4.87 | 3.35, 7.09 |  |
| Z60, Z62-Z65 (Social factors) | 23463(0.23%) | 29(0.93%) | 4.09 | 2.51, 6.65 | 1.37 | 0.82, 2.26 |  |
| **Hospital Bed Size** |  |  |  |  |  |  |  |
| Small | 1788183(17.4%) | 514(16.43%) | 0.94 | 0.81, 1.1 | 1.07 | 0.92, 1.25 |  |
| Medium | 2856124(27.8%) | 895(28.6%) | 1.03 | 0.9, 1.17 | 1.09 | 0.96, 1.24 |  |
| Large | 5631237(54.8%) | 1720(54.97%) | Reference | | Reference | |  |
| **Year of delivery** |  |  |  |  |  |  |  |
| 2016 | 1787548(17.4%) | 659(21.07%) | Reference | | Reference | |  |
| 2017 | 1741778(16.95%) | 605(19.33%) | 0.94 | 0.79, 1.13 | 0.91 | 0.76, 1.09 |  |
| 2018 | 1728127(16.82%) | 564(18.01%) | 0.88 | 0.74, 1.06 | 0.84 | 0.71, 1 |  |
| 2019 | 1702421(16.57%) | 475(15.18%) | 0.76 | 0.63, 0.91 | 0.69 | 0.58, 0.83 |  |
| 2020 | 1673604(16.29%) | 464(14.84%) | 0.75 | 0.62, 0.92 | 0.67 | 0.55, 0.81 |  |
| 2021 | 1642067(15.98%) | 362(11.58%) | 0.6 | 0.49, 0.74 | 0.5 | 0.41, 0.62 |  |

1. Adjusted for demographic, clinical, obstetric, and hospital characteristics. DS indicates data suppressed in accordance with HCUP requirements because the cell size was 10 or fewer.

**Supplemental Table 1. ICD-10 codes used in analysis**

### **Opioid Use Disorder, Dependence, Abuse, and Poisoning**

- F11.10, F11.11, F11.12, F11.13, F11.14, F11.15, F11.159
- F11.20, F11.21, F11.22, F11.23, F11.24, F11.25, F11.259
- F11.90, F11.91, F11.92, F11.93, F11.94, F11.95, F11.959
- T40.0X1A–T40.0X4A (opium poisoning)
- T40.1X1A–T40.1X4A (heroin poisoning)
- T40.2X1A–T40.2X4A (other opioid poisoning)
- T40.3X1A–T40.3X4A (methadone poisoning)
- T40.4X1A–T40.4X4A (synthetic opioid poisoning)

### **Social Determinants of Health (SDOH)**

- **Education/Employment:** Z55.0–Z55.9, Z56.0–Z56.9, Z57.0–Z57.9
- **Physical Environment and Housing:** Z58.0–Z58.9, Z59.0–Z59.9
- **Social and Psychosocial Factors:** Z60.0–Z60.9, Z62.0–Z62.9, Z63.0–Z63.9, Z64.0–Z64.9, Z65.0–Z65.9

### **Mental Health and Substance Use Disorders**

- F32.x–F33.x (depressive disorders)
- F41.x (anxiety disorders)
- F19.x (substance use disorders)

### **Obstetric and Maternal Morbidity Covariates**

- O10.x (chronic hypertension)
- O24.0–O24.3 (pregestational diabetes)
- O99.21x (obesity complicating pregnancy)
- O72.x (postpartum hemorrhage)
- O14.x–O16.x (hypertensive disorders of pregnancy)
- O60.x (preterm labor)
- O36.4xx (intrauterine fetal demise)
- Cesarean delivery: ICD-10-PCS 10D00Z0–10D00Z2
- Third- or fourth-degree perineal laceration: ICD-10-CM O70.2x–O70.3x
- Severe maternal morbidity codes defined per CDC guidelines excluding transfusion
- Pain conditions Schrepf et al.
